# Retraction Note: Efficacy and safety of arthroscopy in femoroacetabular impingement syndrome: a systematic review and meta-analysis of randomized clinical trials

**DOI:** 10.1038/s41598-024-71300-x

**Published:** 2024-09-03

**Authors:** José María Lamo-Espinosa, Gonzalo Mariscal, Jorge Gómez-Álvarez, Mikel San-Julián

**Affiliations:** 1 Hip, Tumors and Pediatric Orthopedic Unit, University Clinic of Navarra, Navarra, Spain; 2 Institute for Research on Muscuoskeletal Disorders, Valencia Catholic University, Carrer de Quevedo, 2, 46001 Valencia, Spain

Retraction of: *Scientific Reports* 10.1038/s41598-023-43441-y, published online 01 October 2023

The Editors have retracted this Article.

After publication concerns were raised regarding the validity of the conclusions drawn. Subsequent post-publication review revealed that due to data errors and use of an incorrect statistical model, the main claim of the paper stating “arthroscopic surgery results in a high rate of conversion to osteoarthritis” can no longer be reliably reached.

José María Lamo-Espinosa, Gonzalo Mariscal, Jorge Gómez-Álvarez & Mikel San-Julián disagree with this retraction.

